# Correlations between serum acute-phase protein SAA, platelet-to-lymphocyte ratio, and disease activity in ankylosing spondylitis

**DOI:** 10.2478/abm-2026-0027

**Published:** 2026-08-31

**Authors:** NingNing Yu, TaiZhe Wang, ZhiGang Liu

**Affiliations:** Department of Pain and Rehabilitation, Wuhan Third Hospital Graduate Joint Training Base, School of Medicine, Wuhan University of Science and Technology, Hubei 430065, China; Orthopedics Department, Shiyan People's Hospital Affiliated to Hubei University of Medicine, Hubei 442000, China; Department of Pain and Rehabilitation, Wuhan Clinical Research Center for Acupotomy Medicine, Wuhan Third Hospital, Hubei 430000, China

**Keywords:** ankylosing spondylitis, biomarker, disease activity, platelet-to-lymphocyte ratio, serum amyloid A

## Abstract

**Background:**

Ankylosing spondylitis (AS) is a chronic inflammatory rheumatic disease characterized by sacroiliitis and spinal osteophyte formation that needs sophisticated testing to improve the accuracy of activity assessment and clinical decision-making.

**Objective:**

To investigate the clinical value of serum amyloid A (SAA) and platelet-to-lymphocyte ratio (PLR) in assessing disease activity in patients with AS.

**Methods:**

Using the bath ankylosing spondylitis disease activity index (BASDAI), 160 AS patients were categorized into 3 subgroups: low activity (BASDAI <4, n = 60), moderate activity (4≤ BASDAI <6, n = 50), and high activity (BASDAI ≥6, n = 50). Quantitative measurements included SAA concentrations and PLR values, with subsequent analysis of their relationship to disease progression. Diagnostic performance was evaluated with receiver operating characteristic (ROC) curve methodology.

**Results:**

The high-activity group showed significantly elevated SAA (48.3 mg/L ± 9.1 mg/L) and PLR (164.5 ± 29.2) versus moderate- (SAA: 29.0 mg/L ± 8.2 mg/L; PLR: 138.4 ± 27.6) and low-activity groups (SAA: 14.3 [11.7–17.2] mg/L; PLR: 102.7 ± 22.2) (all *P* < 0.001). Both markers correlated strongly with BASDAI (SAA: *r* = 0.76; PLR: *r* = 0.71, *P* < 0.001). ROC analysis demonstrated that an SAA cutoff of 30.6 mg/L predicted high activity (96% sensitivity, 80% specificity), while PLR ≥143.7 showed 78.0% sensitivity and 76.4% specificity. Combined detection achieved superior area under the curve (0.95) versus individual markers (SAA: 0.93; PLR: 0.86).

**Conclusion:**

SAA and PLR are strongly associated with BASDAI-defined disease activity in AS, and combined assessment improves identification of high disease activity, supporting their potential role as objective adjunctive markers in clinical assessment.

Ankylosing spondylitis (AS) is a chronic inflammatory rheumatic disease characterized by sacroiliitis and spinal osteophyte formation, with a global prevalence of 0.1%–1.4% [1]. The disease may involve extra-articular manifestations (e.g., psoriasis, uveitis), contributing to reduced quality of life [2,3,4]. Treatment options range from pharmacological/surgical interventions [5, 6] to traditional Chinese therapies (e.g., moxibustion, electroacupuncture) [7, 8]. Although the bath ankylosing spondylitis disease activity index (BASDAI) is the clinical standard, its subjectivity and undefined cutoffs compromise reliability [9,10,11]. Objective biomarkers could address this limitation, enabling earlier diagnosis and tailored interventions.

Serum amyloid A (SAA), an acute-phase protein induced by inflammation, correlates with disease severity [12] and activity in rheumatoid/psoriatic arthritis [13,14,15]. The platelet-to-lymphocyte ratio (PLR), reflecting systemic inflammation, has prognostic value in appendicitis [16], diabetic retinopathy [17], traumatic brain injury [18], and pancreatitis [19]. Notably, PLR also exhibits predictive potential in solid tumors [20, 21]. While prior studies have evaluated inflammatory biomarkers in AS, the incremental value of combining SAA with a routinely available hematologic index such as the PLR for identifying high disease activity remains insufficiently defined. Establishing clinically interpretable cutoffs and performance metrics for a combined strategy may facilitate rapid, objective risk stratification, particularly when standard inflammatory markers yield equivocal results. In clinical practice, SAA testing may be most useful in situations of clinical–laboratory discordance (e.g., high BASDAI with low/normal C-reactive protein [CRP]) or when a combined-marker approach is intended to improve confidence in ruling in high activity. Therefore, this study investigated SAA/PLR correlations with BASDAI-based AS disease activity to test their combined diagnostic utility. The findings may improve the accuracy of activity assessment and clinical decision-making.

## Methods

The present study was approved by the Ethics Committee of The Third Affiliated Hospital of Wuhan University of Science and Technology (No.201903WH90). Written informed consent was obtained from all participants before enrollment. All procedures were performed in accordance with the ethical standards of the Institutional Review Board and the Declaration of Helsinki, and its later amendments or comparable ethical standards.

This single-center prospective study enrolled 160 AS patients diagnosed between March 2021 and March 2024, all of whom met the 1984 modified New York criteria [22]. Exclusion criteria: (1) Patients with concurrent infections, malignancies, or other autoimmune diseases; (2) Patients receiving glucocorticoids or biologic agents within the past year; (3) Pregnant or lactating patients. Participants were stratified into low-activity (n = 60), moderate-activity (n = 50), and high-activity (n = 50) groups based on BASDAI scores. The study was designed as a prospective observational investigation with consecutive recruitment during the study period. A priori formal sample-size calculation was not performed before enrollment. After recruitment, the achieved sample provided adequate precision for receiver operating characteristic (ROC) estimation and correlation analyses, as reflected by the relatively narrow 95% confidence intervals (CIs) for the area under the curves (AUCs). Nevertheless, the absence of a priori sample-size planning should be considered a limitation, and future multicenter studies should predefine the primary endpoint and perform formal power calculations based on expected AUC improvement from adding SAA and PLR to standard markers.

### Grouping and clinical data collection

Disease activity was quantified using the BASDAI [11], a patient-reported questionnaire with scores ranging from 0 to 10 (where higher scores indicate greater disease activity). The stratification was as follows:

Low-activity group: BASDAI <4; Moderate-activity group: 4 ≤BASDAI <6; High-activity group: BASDAI ≥6. Clinical data, including age, disease duration, CRP, and erythrocyte sedimentation rate (ESR), were retrieved from electronic databases, paper-based medical records, and laboratory reports.

### Sample collection

Peripheral venous blood was collected from all AS patients after an overnight fast (≥ 8 h) on the morning after diagnosis. Blood samples were obtained using a vacuum blood collection system (BD Biosciences, USA) and processed as follows: For serum preparation: 3 mL of venous blood was collected in a serum separator tube (red cap), allowed to clot for 30 min, and centrifuged at 3000 rpm for 15 min. For whole blood preparation, 2 mL of venous blood was collected in an ethylenediaminetetraacetic acid dipotassium salt (EDTA-K_2_) anticoagulant tube (purple cap) and gently inverted 8 times to ensure complete mixing. Only fresh samples collected at initial diagnosis were used; no stored or leftover samples were included.

### Laboratory measurements

SAA quantification: Serum from separator tubes was analyzed using a human SAA enzyme-linked immunosorbent assay kit (Shanghai Enzyme-linked Biotechnology, China; Cat. No. ml106609) with duplicate measurements. Absorbance at 450 nm was measured using a microplate reader (BioTek Epoch, USA), with concentrations calculated using a standard curve. Hematological analysis: EDTA-anticoagulated whole blood was analyzed using a Sysmex XN-9000 (Sysmex, Kobe, Japan) automated hematology analyzer to determine both platelet (PLT) and lymphocyte (LYM) counts. The analyzer was calibrated daily with 3-level quality controls. PLR was calculated as PLT (×10^9^/L) divided by LYM (×10^9^/L).

### Statistical analysis

Data were analyzed using Statistical Package for the Social Sciences 26.0 (SPSS Inc., USA). The Shapiro–Wilk test assessed data normality. Normally distributed continuous variables were expressed as mean ± standard deviation and compared using independent Student *t* tests. Non-normally distributed variables were presented as median (interquartile range [IQR]) and analyzed with Mann–Whitney *U* tests. Multiple group comparisons were performed using Kruskal– Wallis *H* tests, with statistically significant PLR values further analyzed by Dunn–Bonferroni post hoc tests. Categorical data were reported as frequencies (percentages) and analyzed using chi-square or Fisher exact tests. ROC curve analysis evaluated the diagnostic value of SAA and PLR, with optimal cut-off values determined by Youden's index and corresponding sensitivity/specificity calculated. For combined detection, a binary logistic regression model including SAA and PLR was fitted, and the predicted probability was used to generate the ROC curve. Spearman's rank correlation analyzed associations between non-parametric variables. The significance level was set at α = 0.05, with *P*-values <0.05 considered statistically significant.

## Results

### Clinical baseline characteristics

A cohort of 160 individuals diagnosed with AS was prospectively evaluated between March 2021 and March 2024. The study population demonstrated the following clinical parameters: age range 21–59 years, body mass 38–106 kg, CRP levels 2.7–46.1 mg/L, and ESR 6–56 mm/h. Comparative analysis revealed that patients classified into the high disease activity category showed markedly increased concentrations of both CRP and ESR (*P* < 0.001), thereby validating the BASDAI stratification approach (**Table 1**). Both SAA and PLR were significantly higher in the high-activity group than in moderate- and low-activity groups.

1. SAA: Significantly higher in high-activity group vs. moderate/low groups (analysis of variance, *P* < 0.001).
2. PLR: Significantly elevated in the high-activity group (Kruskal–Wallis test, *P* < 0.001).
3. Data presentation: Normally distributed data: Mean ± SD Non-normally distributed data: Median (IQR).
4. Group comparisons: CRP and ESR were significantly higher in the high-activity group (*P* < 0.001), supporting BASDAI classification validity.

**Table 1.** Baseline demographic and clinical characteristics of AS patients by BASDAI activity group

**Group**	**N**	**Age (years)**	**Weight (kg)**	**CRP (mg/L)**	**ESR (mm/h)**	**PLR**	**SAA (mg/L)**	**BASDAI**
Low	60	34.5 (26.8–44.2)	66 ± 8.7	7.4 (4.7–16.3)	12 (10–16)	102.7 ± 22.2	14.3 (11.7–17.2)	2.8 (2.1–3.1)
Moderate	50	42 (35.2–47.8)	69 (63–80)	16.1 (12.1–21.2)	24.5 (21–30)	138.4 ± 27.6	29 ± 8.2	4.7 ± 0.4
High	50	45.5 (36.2–51.8)	73.4 ± 14	22 (13.8–31.8)	40.5 (31–49)	164.5 ± 29.2	48.3 ± 9.1	6.8 (6.5–7.4)

AS, ankylosing spondylitis, BASDAI, bath ankylosing spondylitis disease activity index; CRP, C-reactive protein; ESR, erythrocyte sedimentation rate; PLR, platelet-to-lymphocyte ratio; SAA, serum amyloid A.

### Comparison of SAA and PLR levels

Serum SAA levels were significantly higher in the high-activity group (48.3 ± 9.1 mg/L) than in the moderate- (29.0 mg/L ± 8.2 mg/L) and low-activity groups (14.3 [11.7–17.2] mg/L) (**Figure 1A**). Similarly, PLR was signi-ficantly elevated in the high-activity group (164.5 ± 29.2) compared to the moderate- (138.4 ± 27.6) and low-activity groups (102.7 ± 22.2) (**Figure 1B**) (all *P* < 0.001).

**Figure 1.**
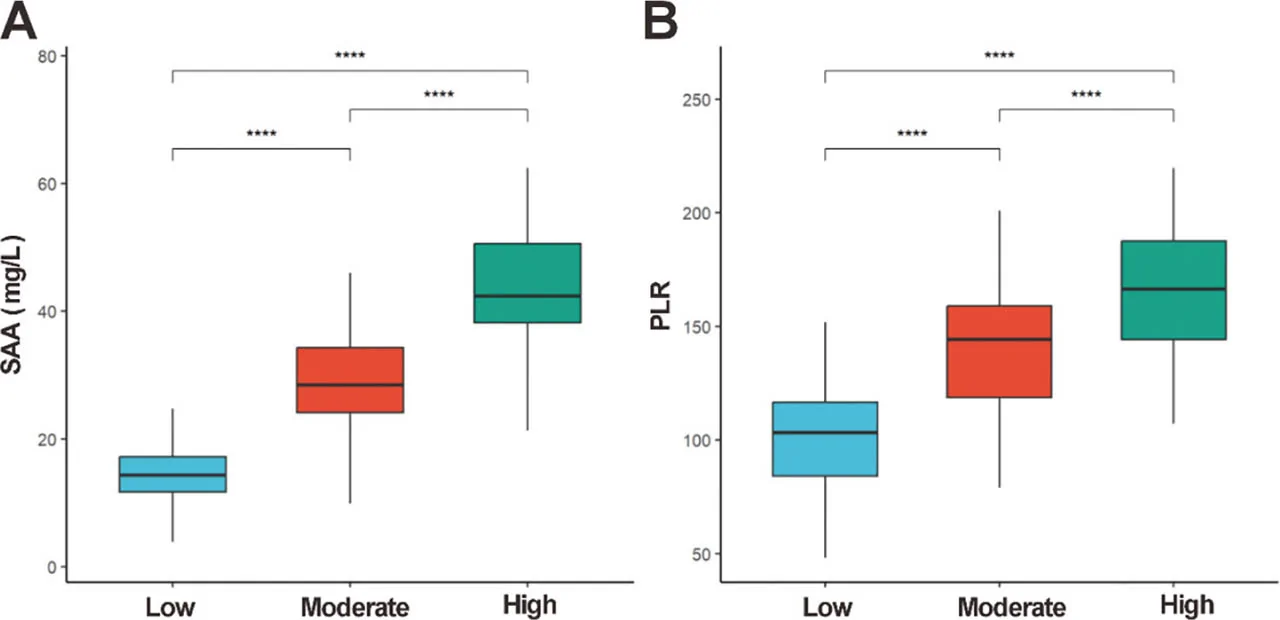
Distribution of SAA and PLR. **(A)** SAA; **(B)** PLR levels across different disease activity groups. Box plots display medians (horizontal lines), IQR (boxes), and 1.5 × IQR whiskers. Group comparisons were analyzed by Kruskal–Wallis H test, with post hoc Dunn–Bonferroni tests for significant PLR differences: low- vs. high-activity (*P* < 0.0001), moderate- vs. high-activity (*P* < 0.0001), and low- vs. moderate-activity (^∗∗∗∗^*P* < 0.0001). IQR, interquartile range; PLR, platelet-to-lymphocyte ratio; SAA, serum amyloid A.

### Correlation analysis of SAA, PLR and BASDAI scores

Spearman correlation analysis showed strong positive correlations between SAA and BASDAI (*r* = 0.76, *P* < 0.001) and between PLR and BASDAI (*r* = 0.71, *P* < 0.001) (**Figures 2A,B**). For combined detection, predicted probabilities from a binary logistic regression model including SAA and PLR were used to construct the ROC curve (**Table 2**). A moderate positive correlation was observed between SAA and PLR (*r* = 0.54, *P* < 0.001) (**Figure 2C**).

**Figure 2.**
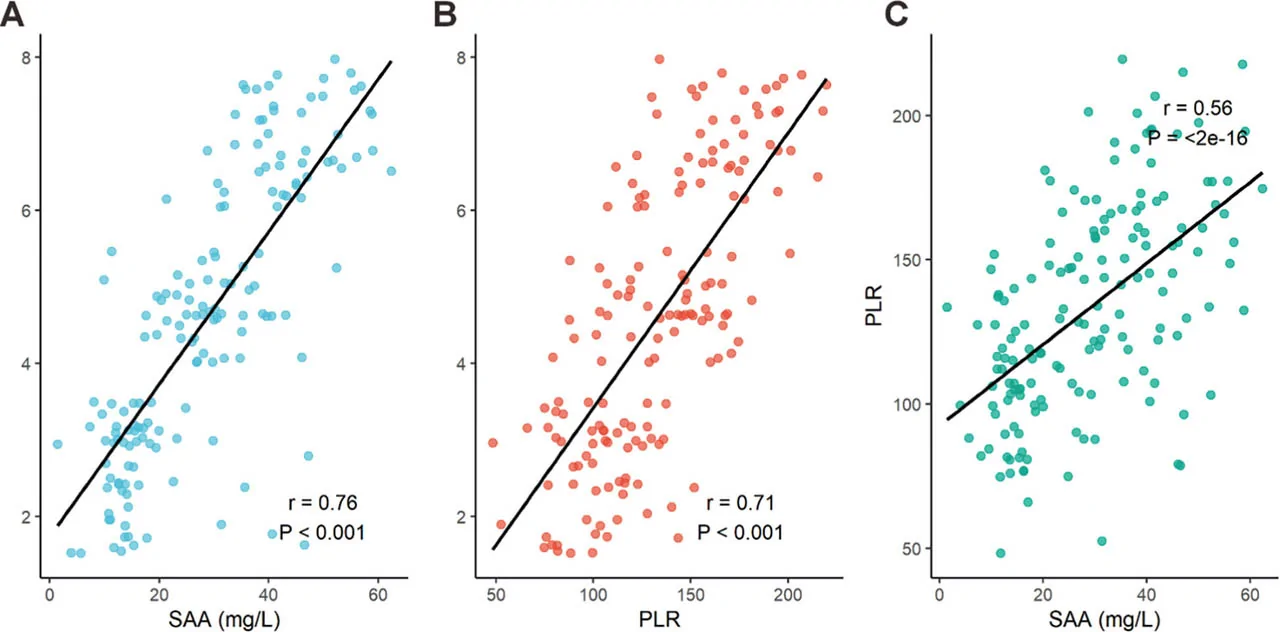
Correlation of SAA, PLR and BASDAI scores. **(A)** SAA and BASDAI; **(B)** PLR and BASDAI, and **(C)** SAA and PLR. Both SAA and PLR showed strong correlations with BASDAI, while SAA and PLR exhibited a moderate correlation. r: correlation coefficient; *P* < 0.001 for all. BASDAI, bath ankylosing spondylitis disease activity index; PLR, platelet-to-lymphocyte ratio; SAA, serum amyloid A.

**Table 2.** ROC curve analysis of diagnostic performance

**Cut-off value**	**Sensitivity (%)**	**Specificity (%)**	**AUC**
SAA ≥30.6 mg/L	96.0	80.0	0.93
PLR ≥143.7	78.0	76.4	0.86
Combined detection	86.0	90.9	0.95

AUC, area under the curve; PLR, platelet-to-lymphocyte ratio; ROC, receiver operating characteristic; SAA, serum amyloid A.

### Diagnostic value of SAA and PLR for AS

ROC curve analysis revealed that SAA ≥30.6 mg/L predicted high disease activity with an AUC of 0.93 (95% CI: 0.89–0.97), sensitivity 96.0%, and specificity 80.0%. PLR ≥143.7 had an AUC of 0.86 (95% CI: 0.82–0.91), sensitivity 78.0%, and specificity 76.4%. Combined SAA + PLR improved diagnostic performance (AUC = 0.95, 95% CI: 0.89–0.99), with 86.0% sensitivity and 90.9% specificity (**Figure 3** and **Table 2**).

**Figure 3.**
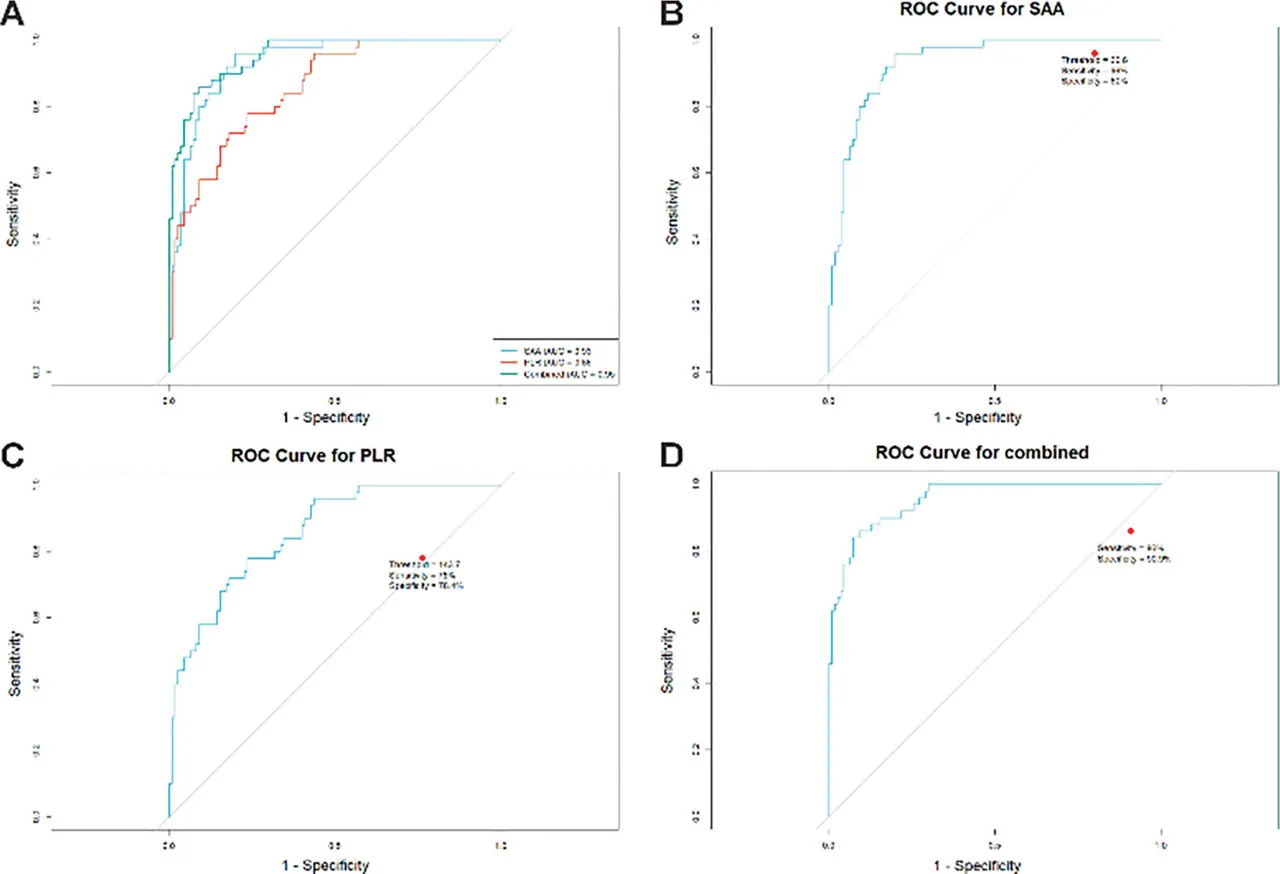
ROC curve analysis of SAA, PLR, and their combination for predicting high disease activity. **(A)** AUC comparison between SAA and PLR; **(B)** sensitivity/specificity of SAA; **(C)** sensitivity/specificity of PLR; **(D)** sensitivity/specificity of combined SAA + PLR. AUC, area under the curve; combination, SAA + PLR; PLR, platelet-to-lymphocyte ratio; ROC, receiver operating characteristic; SAA, serum amyloid A.

## Discussion

In this study, SAA and PLR increased stepwise across BASDAI-defined low-, moderate-, and high-activity groups, and both markers were strongly correlated with BASDAI. SAA demonstrated excellent discrimination for high disease activity, and combining SAA with PLR further improved diagnostic performance compared with either marker alone. These findings support the potential utility of combining an acute-phase reactant (SAA) with a readily obtainable hematologic inflammatory index (PLR) as objective adjuncts for disease activity assessment in AS.

Emerging evidence suggests that sustained inflammatory microenvironments in AS may promote oxidative stress and epigenetic dysregulation [23, 24]. SAA, a hepatocyte-derived acute-phase protein regulated by interleukin 6/tumor necrosis factor-α (TNF-α), can increase 1,000-fold within 4–6 h post-inflammation [25]. In clinical settings, CRP remains the most widely available and cost-effective acute-phase biomarker in AS. However, a subset of patients may exhibit discordance between clinical activity and CRP/ESR, and acute-phase proteins differ in kinetics and regulatory pathways. SAA is a rapidly responsive acute-phase reactant and may provide complementary information regarding systemic inflammatory burden, particularly in patients with high symptom activity but relatively modest CRP elevations. In this context, SAA may be considered an adjunct rather than a substitute for CRP, potentially improving identification of high-activity states when used in combination with hematologic inflammatory indices such as PLR. Elevated SAA may directly mirror systemic inflammatory burden in AS, potentially via nuclear factor kappa-light-chain-enhancer of activated B cells activation and matrix metalloproteinase-induced cartilage degradation [26, 27]. The strong correlation between SAA and BASDAI (*r* = 0.76, *P* < 0.01) aligns with prior findings linking SAA to sacroiliitis severity on magnetic resonance imaging [28] and its utility in spondyloarthritis progression prediction [29]. The PLR, a composite biomarker of systemic inflammation [30], demonstrated significant correlation with BASDAI scores (*r* = 0.71, *P* < 0.01). This reflects its dual pathological basis: platelet-derived cytokine release (proinflammatory) and lymphopenia-associated immune dysregulation. Compared to conventional markers (CRP and ESR), PLR showed superior discriminative capacity for high disease activity (AUC = 0.86). Although PLR's role in musculoskeletal disorders is recognized [31], its application in AS remains underexplored. Based on our findings, SAA and PLR may serve as adjunctive markers to support identification of high-activity states, particularly when clinical symptoms are prominent, but CRP is inconclusive. A pragmatic approach could be to consider high activity more likely when SAA ≥30.6 mg/L and PLR ≥143.7, while recognizing that thresholds require external validation and should not replace comprehensive clinical assessment. Future studies should investigate integrating SAA and PLR into comprehensive AS activity assessments to reduce reliance on subjective scoring.

The limitations of standalone biomarkers (e.g., CRP's variable sensitivity due to genetic polymorphisms [32, 33]) highlight the necessity for multidimensional assessment strategies. ROC analysis demonstrated that the combination of SAA and PLR achieved superior diagnostic performance (AUC = 0.95, 95% CI: 0.89–0.99) compared to individual markers (SAA: AUC = 0.93; PLR: AUC = 0.86). This synergistic effect likely arises from their complementary biological roles: SAA primarily reflects acute-phase, IL-6-driven inflammation, whereas PLR indicates chronic, TNF-α-mediated immune dysregulation—thereby collectively addressing AS heterogeneity. These findings align with Liuet al.'s reports in community-acquired pneumonia [34]. Notably, the SAA/PLR combination has demonstrated prognostic value in other pathologies, including colorectal cancer staging [35], suggesting broader applications for early detection and treatment assessment.

This single-center, cross-sectional study cannot establish causality or assess within-patient changes in SAA/PLR over time or in response to treatment. The absence of a control group limits contextual interpretation of absolute biomarker values, and excluding patients who recently received biologics or glucocorticoids may have introduced spectrum bias and reduced generalizability to more severe or treatment-refractory AS. In addition, PLR—derived from PLT and LYM counts—may be influenced by factors that affect hematologic indices or systemic inflammation (e.g., non-steroidal anti-inflammatory drug, conventional synthetic disease-modifying anti-rheumatic drugs, other immunomodulatory agents) as well as comorbidities; therefore, residual confounding from concomitant medications and unmeasured clinical variables cannot be fully excluded.

## Conclusion

SAA and PLR were strongly associated with BASDAI-defined disease activity in AS, with both markers increasing across low-, moderate-, and high-activity groups. SAA showed excellent discrimination for high disease activity, and combining SAA with PLR improved diagnostic performance compared with either marker alone. These readily obtainable indices may serve as practical adjunctive markers for objective assessment of disease activity, although external validation and longitudinal studies are required before broad clinical implementation.
